# Low prevalence of the transmitted HIV-1 drug resistance among newly diagnosed HIV-1 individuals in Jiangsu Province, China during 2009–2011

**DOI:** 10.1186/s12889-015-1489-8

**Published:** 2015-02-10

**Authors:** Hongxiong Guo, Xiaoqin Xu, Haiyang Hu, Ying Zhou, Haitao Yang, Tao Qiu, Gengfeng Fu, Xiping Huan

**Affiliations:** Jiangsu Provincial Center for Disease Control and Prevention, 172 Jiangsu Road, Nanjing, 210009 China

**Keywords:** HIV-1, The transmitted HIV drug resistance mutation, Subtype

## Abstract

**Background:**

Prevalence of The transmitted HIV drug resistance (THDR) has been reported in many countries. In China, the low level THDR was found in only a few provinces. To know the transmitted HIV drug resistance in east of China, we investigated THDR during 2009–2011 in Jiangsu province of China.

**Methods:**

Between January and August of 2009, 2010, and 2011, we consecutively collected 50, 54, 53 blood specimens respectively from qualified individuals at surveillance sentinel sites in Jiangsu province according to protocol of HIV Drug Resistance Threshold Survey (HIVDR-TS) recommended by WHO. The region of *pol* gene including protease and partial retro-transcriptase was amplified, sequenced and edited. Then the sequences were submitted to HIV drug resistance database to analysis transmitted HIV drug resistance mutations using Calibrated Population Resistance tool. The reference sequences of different HIV-1 subtypes were downloaded from HIV database and Genebank. The phylogenetic trees were inferred using the neighbor-joining method.

**Results:**

Our results show that THDR has been at low level from 2009 to 2011, only K101E and V179D mutation was detected which did not belong to the major HIV-1 drug resistance mutations. Phylogenetic analysis showed that CRF01_AE is the predominant subtype, and followed by CRF07_BC and B subtype. Subtype B consists of the two distinct clusters.

**Conclusions:**

The low level of THDR suggests that anti-retroviral treatment was implemented more effectively and THDR surveillance should be conducted two years later in Jiangsu province of China. CRF01_AE has become the predominant subtype and dual infection of HIV may be common in Jiangsu province.

## Background

By the end of 2011, it was estimated that more than 780,000 people lived with HIV-1 and a cumulative total of 157,050 AIDS patients had received antiretroviral therapy in China [1]. In order to curb rapid HIV transmission and improve the quality of life for people living with HIV, the national level antivirus treatment program began in 2002 [2]. During the first two years of the program, the prevalence of HIV drug resistance among AIDS patients on anti-retroviral treat (ART) for six months reached to 62.7% [3]. Furthermore, some studies showed that the HIV-1 drug resistance among naive AIDS patients ranged from 2.8% to20.2% in China [4-8]. Currently, the most popular HAART regimens consist of six drugs which are lamivudine (3TC), Stavudine (D4T), Efavirenz (EFV), Nevirapine (NVP), Zidovudine (AZT) and Tenofovir (TDF) [9]. Some normal 2^nd^ line drugs such as tenofovir(TDF) and lopinavir/ritonavir (LPV/r), were introduced in 2008 but had not been used widely. The transmission of HIV drug resistance strains is common in the majority of countries with widely used ART, especially in developing countries with limited resources [10-13]. Recently, transmitted HIV drug resistance has been found in a few provinces where ART was introduced earlier in China [9,14-16].

Jiangsu province, located in the east of China, is an economically developed region with a population of 80 million. The first AIDS case was recorded in 1991 in Jiangsu, and a total of 5471 cases were reported by the end of 2011. The Free ART project was initiated in Jiangsu province in 2005. Up to now, more than 4000 AIDS patients have received free ART. After seven years of the promotion of free ARF in Jiangsu province, the prevalence of drug resistance strain among AIDS patients receiving ART reached to 8.5% [17]. However, it is unclear whether the transmitted HIV drug resistance has begun to spread in Jiangsu Province.

To further investigate the prevalence of the transmitted of HIVDR, we conducted the threshold survey of the transmitted HIV drug resistance among naïve patients according to the protocol which was recommend by World Health Organization (WHO)from 2009 to 2011 [18]. In this study, we report the estimated prevalence of HIVDR transmission and subtypes of transmitted HIVDR in Jiangsu province of China.

## Methods

Between January and August of 2009, 2010, and 2011, we consecutively collected 50, 54, 53 blood specimens, respectively from qualified individuals at surveillance sentinel sites in Jiangsu province according to HIV Drug Resistance Threshold Survey(HIVDR-TS)by the WHO for the surveillance of HIVDR in countries with limited resources. This study was approved by Ethics Committee of Jiangsu Provincial Center for Disease Prevention and Control. The written informed consent from participants was obtained after they knew the objective and content of this study. No participants less than 18 years old were involved in this study.

The eligible individuals were recruited according to HIV Drug Resistance Threshold Survey(HIVDR-TS)by the WHO for the surveillance of HIVDR in countries with limited resources [18-20]. According to the WHO HIVDR-TS protocol, 47 to 50 qualified sequences obtained from consecutively collected HIV-positive specimens are required to determine the level of the transmitted HIVDR. All eligible individuals were under age 25 years at HIV diagnosis and no pregnancy history for females.

A total of 5 mL of whole blood was collected into EDTA-K3 vacuum tubes. Plasma samples were obtained by centrifugation and aliquots were stored at −70°C. Viral RNA was extracted from 140 μL plasma using the QIAamp Viral RNA Mini kit (Qiagen, Gmbh, Hilden, Germany) according to the manufacturer’s instruction. The 1300 bp *pol* region of HIV-1 includes reverse transcription polymerase and protease gene was amplified with the previous described methods [21]. The viral RNA was amplified in an one step reverse transcription chain reaction using SuperScript®IIIone-step RT-PCR with platinum®Taq kit (Invitrogen, Carlsbad, CA, USA), followed by a second round PCR using Platinum®TaqPCRx DNA Ploymerase kit (Invitrogen, Carlsbad, CA, USA). The obtained fragments were purified with gel extraction kit (Qiagen, Gmbh, Hilden, German), and then sequenced in 3730X DNA Analyzer System.

To eliminate potential laboratory contamination, all sequences were first subjected to an HIV-1 Blast search to compare with related reference sequences in the HIV Databases sponsored of the Division of AIDS of the National Institute of Allergy and Infectious of AIDS (NIAID), The National Institute of Health (NIH) (http://www.ncbi.nlm.nih.gov/). Nucleotide sequences were aligned with the references using Clustal W algorithm of MAGE 5.0. Alignment quality, then were checked manually in Bioedit. The protease and retro-transcriptase mutation were interpreted using the Calibrated Population Resistance (CPR) tool according to the WHO list for determination of transmitted HIV-1 DR (http://cpr.standard.edu/cpr.cgi).

HIV-1 subtypes reference sequences were downloaded from HIV database (http://www.hiv.lanl.gov/content/sequence/HIV/mainpage.html). HIV sequences reported in China were downloaded from Genebank (http://www.ncbi.nlm.nih.gov/). The phylogenetic trees were inferred using the neighbor-joining method. All positions containing gaps and missing data were eliminated from the dataset. Phylogenetic analyses were conducted in MEGA 5.0.

The GenBank accession numbers of the HIV-1 sequences reported in this article are KJ724119 - KJ724248.

## Results

### The social-demographic data of newly diagnosed HIV-1 positive people

Background information on this newly diagnosed HIV-1 positive population is summarized in Table 1. Among them, 128 out of 146 (87.7%) were male, and 124 (84.9%) were single; 140 (95.9%) belongs to the Han ethnic group. As to educational level of them, 44 (30.1%) received the education of middle school level, 39 (26.7%) finished high school, and 56 (28.4%) have studied at least college. There were 31 subjects aged 24 years old (21.2%) and 28 (19.2%) aged 25 years old. MSM activity (56.8%) was the main infection route, followed by heterosexual route (39.1%).

**Table 1:** **The characteristics of HIV-1 positive individuals and subtypes circulating among newly diagnosed HIV-1 positive population less than 25 years old (N = 146)**

**Characteristics**	**N (%)**
Sex	
*Male*	128(87.7)
*Female*	18(12.3)
Marital status	
*Single*	124(84.9)
*Married*	22(15.1)
Race/Ethnicity	
*Chinese*	140(95.9)
*Yi*	4(2.7)
*Zhuang*	1(0.7)
*Tujia*	1(0.7)
Birth province	
*Jiangsu*	142(97.3)
*Other provinces*	4(2.7)
Education	
*Primary school*	7(4.8)
*Middle school*	44(30.1)
*High middle school*	39(26.7)
*College or higher*	56(28.4)
Age	
*Less than 20*	10(6.8)
*20*	13(8.9)
*21*	24(16.4)
*22*	26(17.8)
*23*	14(9.6)
*24*	31(21.2)
*25*	28(19.2)
Infectious route	
*MSM*	83(56.8)
*Heterosexual*	57(39.1)
*IDU*	4(2.7)
*Not determination*	2(1.4)
Subtypes	
*CRF01_AE*	78(53.4)
*B*	18(12.3)
*CRF07_BC*	29(19.9)
*CRF08_BC*	7(4.8)
*C*	1(0.7)
*URF*	13(8.9)

### The mutation related to drug resistance

In this study, we were able to consecutively genotype 47 of 50 serum specimens (94%) in 2009, 50 of 54 (92.6%) in 2010, 49 of 53 (92.5%) in 2011. As shown in Table 2, the prevalence of transmitted HIV drug resistance was 2.1% (1/47) in 2009, 4.0%(2/50) in 2010, and 4.1%(2/49) in 2011, respectively. Only two mutations associated with DR were found. They are K101E and V179D mutation. K101E mutation can cause intermediate resistance to NVP and delairdine (DLV) and low-level resistance to EFV and etravirine (ETR). V179D mutation is a borderline/suspicious mutation associated with transmitted HIVDR can reduce the susceptibility of NVP, EFV, and ETR against HIV. But no major mutation related to NRTI or NNRTI were found. No mutation related to PI was also found.

**Table 2:** **The mutations related to HIV-1 drug resistance among newly diagnosed HIVcp1 positive population less than 25 years old**

**Mutation**	**Year**		
	**2009**	**2010**	**2011**
V179D	1(2.1%)	1(2.0%)	1(2.0%)
K101E	0(0.0%)	1(2.0%)	1(2.0%)

### Phylogenetic analysis

**Figure 1:**
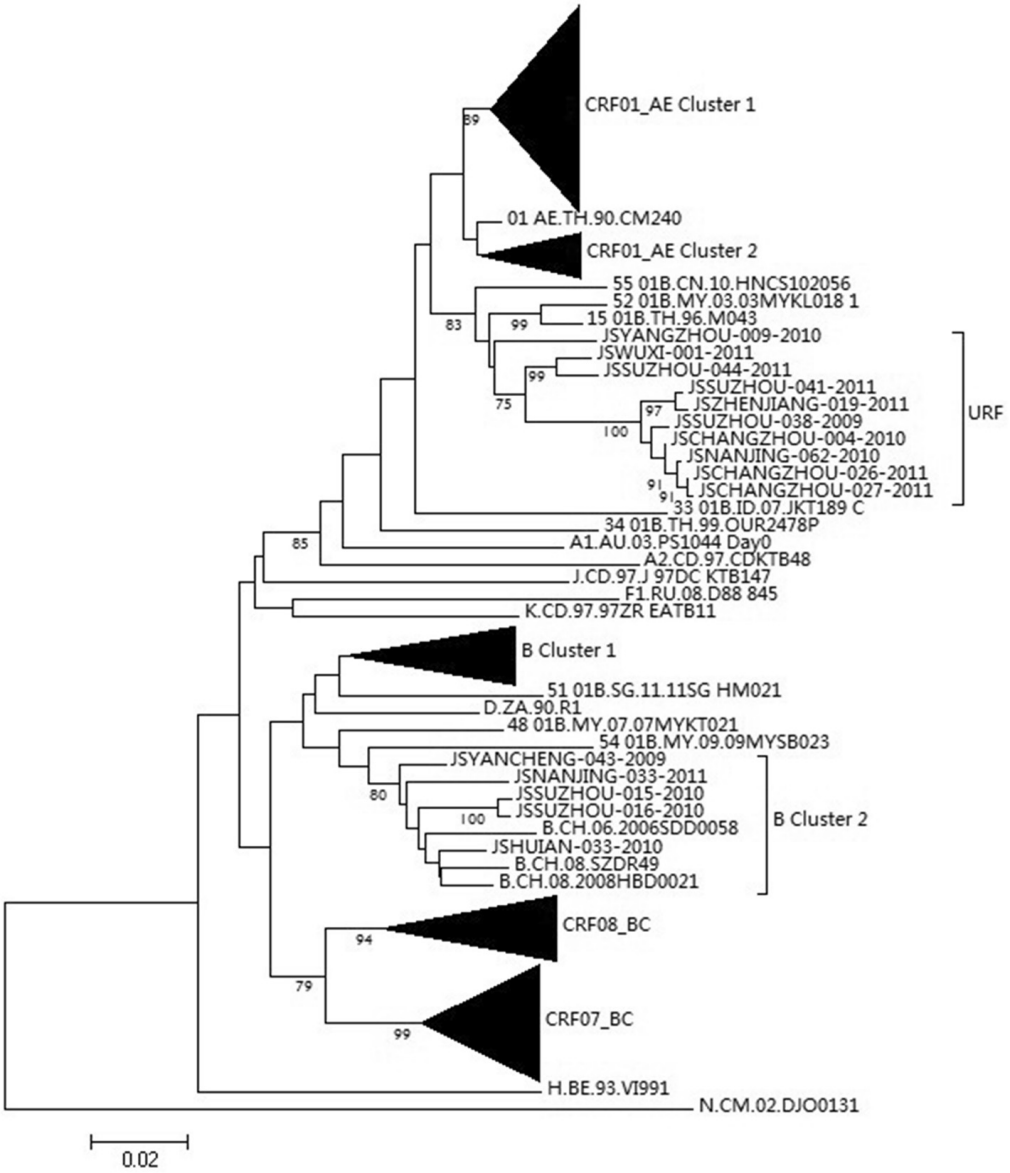
**The phylogenetic tree based on partial** ***pol*** **gene fragment of HIV-1.** The tree topologies were inferred by the neighbor-joining method. Numbers at the nodes represent the percentages of bootstrap values (only values > 75% are shown).

Based on sequence of the fragment of Protease and partial reverse transcriptase of *pol* gene, we analyzed the subtypes of HIV-1 circulating in newly diagnosed HIV-1 positive population. CRF01_AE was predominant, accounting for 56.8%, followed by CRF07_BC (19.9%). B subtype only reached to 12.3%. CRF08_BC was 7.8% and only one belonged to C subtype. Unique recombinant forms (URF) accounted for 8.9% of them. As showed in Figure 1, two clusters designated cluster 1 and 2 were observed among B and URFs sequences in phylogenetic tree analysis, respectively.

## Discussion

This is the first study that describes the prevalence of transmitted HIVDR in the southeast of China. It was found that the prevalence of transmitted HIVDR was less than 5%. Moreover, no major HIVDR mutations were found. The level of transmitted HIVDR was low. Surveillance may be repeated two years later according to WHO recommendation [20]. The prevalence of transmitted HIVDR in Jiangsu province is similar to that reported in other regions other than Dehong Prefecture in Yunnan province where the transmitted HIV reached medial level [14,21]. However, the major mutation related HIVDR were found in Henan and Hunan provinces where ART was launched earlier and HIV and HIVDR prevalence were higher in these two provinces [21].

In this study, 95.9% were infected with HIV via sexual transmission routes. MSM has exceeded the heterosexual transmission route. It may indicate in the near future, MSM will become the main transmission route in Jiangsu province. It was partially supported by our unpublished surveillance data that the number of HIV positive people via MSM was close to those through heterosexual behavior among all HIV-1 positive people reported in recent three years.

The change of the transmission route and HIV high risk group often synchronized with the changes of HIV subtypes distribution, followed by the complexity of HIV virus in this region. In this study, CRF01_AE was the predominated subtype. However, CRF07_BC/08_BC (36.8%) had been the major subtypes circulating in Jiangsu province, followed by CRF01_AE (31.6%) before 2007 [22]. MSM played a very important role in making this change. Of course, these are other factors contributing to this change. For example, in previous study, the ages of subjects ranged from 3 to 56, and transmission routes included heterosexual routine, MSM, blood donors, blood transfusion, intravenous drug users and mother to child transmission. Nonetheless, as the predominant subtype in young people, CRF01_AE will remain as the main subtype circulating in Jiangsu Province in the coming years.

As shown in Figure 1, B subtypes circulating in Jiangsu Province consists of two clusters. In Jiangsu Provinces, B subtypes has different origin. In early phase of HIV prevalence in Jiangsu Province, blood donor and blood transfusion was the major transmission route. Among blood donors and patients having experienced blood transfusion, B subtype has been the predominant, these B subtypes were introduced into Jiangsu Province from Henan Province which is bordered the north of Jiangsu Province [22]. The other B subtypes originated from Thailand B in heterosexual transmission group or from Europe-America B in men who sex with men.

It is common that MSM often have many sexual partners and unproductive anal intercourse in China [23]. The sexual abuse among this population will cause HIV dual infection and super infection. Therefore, recombination between intra and inter subtypes was formed. As shown in Figure 1, URFs sequence reached to 8.9%. It implied that HIV dual infection was common in this population and it was supported by the fact that all these URFs sequences are from MSM. In addition, two URFs clusters suggested that these URFs had different origins and the recombinant phenomenon may be common in this population.

## Conclusion

It is the first time to report the prevalence of transmitted HIVDR viruses in Jiangsu province, although the prevalence of transmitted HIVDR in the newly diagnosed HIV-infected population is low. It is critical to continue the HIVDR surveillance after the promotion of ART therapy. Our results also indicate that CRF01_AE has become the predominated subtype and dual infection of HIV may be common in Jiangsu province. The control of the sexual transmission of HIV-1 includes MSM and heterosexual activities would result in decreasing HIV epidemic in Jiangsu province, China.

## Abbreviations

HIV: Human immunodeficiency virus
THDR: The transmitted HIV drug resistance
HIVDR: HIV drug resistance
URF: Unique recombinant form
ART: Anti-retroviral treatment
WHO: World Health Organization
HIVDR-TS: HIV drug resistance threshold survey
MSM: Men who sex with men
PI: Protease inhibitor

## References

[1] State Council AIDS Working Committee Office & UN Theme Group on AIDS in China (2011). A joint assessment of HIV/AIDS Prevention, treatment and care in China (2009).

[2] Zhang F, Dou Z, Ma Y, Zhao Y, Liu Z, Bulterys M (2009). Five-year outcomes of the China National Free Antiretroviral Treatment Program. Ann Intern Med.

[3] Zhang F, Dou Z, Ma Y, Zhang Y, Zhao Y, Zhao D (2011). Effect of earlier initiation of antiretroviral treatment and increased treatment coverage on HIV-related mortality in China: a national observational cohort study. Lancet Infect Dis.

[4] Li J, Li H, Li L, Li H, Wang Z, Yang K (2005). Prevalence and evolution of drug resistance HIV-1 variants in Henan, China. Cell Res.

[5] Huang D, Li Y, Tan W, Zheng C, Zhang Y, Gan Y (2013). Surveillance of primary drug resistance gene mutation for HIV infected men who have sex with men in Shenzhen in 2010. Mod Prev Med.

[6] Xie M, Yan Y, Yan P, Wu S, Chen G, Zhang C (2013). Primary drug-resistant gene mutation to HIV-1 infected MSM in Fujian Province. Chinese J Zoonoses.

[7] Han X, Zhao B, Sun F, An M, Yin L, Zhang H (2012). Primary HIV-1 drug resistance among injecting drug users in Xinjiang. Chinese J Public Health.

[8] Su X, Zhi J, Han J, Wu Y, Li X, Pei L (2012). HIV-1 subtypes and primary drug resistance among recently infected men who have sex with men in China. Int J Virol.

[9] Xing H, Wang X, Liao L, Ma Y, Su B, Fu J (2013). HIV drug resistance and its impact on antiretroviral therapy in Chinese HIV-infected patients. Plos One.

[10] Ndembi N, Lyagoba F, Nanteza B, Kushemererwa G, Serwanga J, Katongole-Mbidde E (2008). Transmitted antiretroviral drug resistance surveillance among newly HIV type 1-diagnosed women attending an antenatal clinic in Entebbe, Uganda. AIDS Res Hum Retroviruses.

[11] Ndembi N, Abraha A, Pilch H, Ichimura H, Mbanya D, Kaptue L (2008). Molecular characterization of human immunodeficiency virus type 1 (HIV-1) and HIV-2 in Yaoundé, Cameroon: evidence of major drug resistance mutations in newly diagnosed patients infected with subtypes other than subtype B. J Clin Microbiol.

[12] Smith DM, Wong JK, Shao H, Hightower GK, Mai SH, Moreno JM (2007). Long-term persistence of transmitted HIV drug resistance in male genital tract secretions: implications for secondary transmission. J Infect Dis.

[13] Hurt CB, McCoy SI, Kuruc J, Nelson JA, Kerkau M, Fiscus S (2009). Transmitted antiretroviral drug resistance among acute and recent HIV infections in North Carolina, 1998 to 2007. Antivir Ther.

[14] Chen X, Xing H, Zhen J, Zhou S, Ruan Y, Qin B (2008). Study on the threshold of HIV-1 drug resistance in Hunan province. Chinese J Epidemiol.

[15] Yuan Y, Cao X, Liu H, Xing H, Liu C, Cui W (2009). Study on the transmission of drug resistant human immunodeficiency virus-1 in Henan province. China J Prev Med.

[16] Zhang J, Kang D, Lin B, Sun X, Fu J, Bi Z (2012). HIV type 1 virological response and prevalence of HIV type 1 drug resistance among patients receiving antiretroviral therapy, Shandong, China. AIDS Res Hum Retroviruses.

[17] Xiao Z, Guo H, Fu GH, Li L, Xu X, Yang C (2011). Mutation of drug resistant gene in HIV infected patients with antiretroviral therapy in Jiangsu province. China J Public Health.

[18] World Health Organization. World Health Organization Global Strategy for the Surveillance and Monitoring of HIV Drug Resistance. http://apps.who.int/iris/bitstream/10665/77349/1/9789241504768_eng.pdf.

[19] Bennett DE, Myatt M, Bertagnolio S, Sutherland D, Gilks CF (2008). Recommendations for surveillance of transmitted HIV drug resistance in countries scaling up antiretroviral treatment. Antivir Ther.

[20] Myatt M, Bennett DE (2008). A novel sequential sampling technique for the surveillance of transmitted HIV drug resistance by cross-sectional survey for use in low resource settings. Antivir Ther.

[21] Liao L, Xing H, Shang H, Li J, Zhong P, Cheng H (2010). The prevalence of transmitted antiretroviral drug resistance in treatment naïve HIV-infected individuals in China. J Acquir Immune Defic Syndr.

[22] Su B, Liu L, Wang F, Gui X, Zhao M, Tien P (2003). HIV-1 subtype B’ dictates the AIDS epidemic among paid blood donors in the Henan and Hubei provinces of China. AIDS.

[23] Hao C, Lau JT, Zhao X, Yang H, Huan X, Yan H (2014). Associations between perceived characteristics of the peer social network involving significant others and risk of HIV transmission among men who have sex with men in China. AIDS Behav.

